# Bilateral Intracranial Beta Activity During Forced and Spontaneous Movements in a 6-OHDA Hemi-PD Rat Model

**DOI:** 10.3389/fnins.2021.700672

**Published:** 2021-08-12

**Authors:** Soheil Mottaghi, Sandra Kohl, Dirk Biemann, Samuel Liebana, Ruth Eneida Montaño Crespo, Oliver Buchholz, Mareike Wilson, Carolin Klaus, Michelle Uchenik, Christian Münkel, Robert Schmidt, Ulrich G. Hofmann

**Affiliations:** ^1^Neuroelectronic Systems, Department of Neurosurgery, Medical Center, University of Freiburg, Freiburg, Germany; ^2^Faculty of Medicine, University of Freiburg, Freiburg, Germany; ^3^Technical Faculty, University of Freiburg, Freiburg, Germany; ^4^Department of Engineering, University of Cambridge, Cambridge, United Kingdom; ^5^Department of Physiology, Anatomy, and Genetics, University of Oxford, Oxford, United Kingdom; ^6^Biomedical Department, Faculty of Engineering, University of Minnesota, Minneapolis, MN, United States; ^7^Department of Psychology, The University of Sheffield, Sheffield, United Kingdom

**Keywords:** 6-hydroxydopamine, Hemi Parkinson’s, deep brain stimulation, animal model, neuroprosthetic, local field potential, beta oscillation, biomarker

## Abstract

Cortico-basal ganglia beta oscillations (13–30 Hz) are assumed to be involved in motor impairments in Parkinson’s Disease (PD), especially in bradykinesia and rigidity. Various studies have utilized the unilateral 6-hydroxydopamine (6-OHDA) rat PD model to further investigate PD and test novel treatments. However, a detailed behavioral and electrophysiological characterization of the model, including analyses of popular PD treatments such as DBS, has not been documented in the literature. We hence challenged the 6-OHDA rat hemi-PD model with a series of experiments (i.e., cylinder test, open field test, and rotarod test) aimed at assessing the motor impairments, analyzing the effects of Deep Brain Stimulation (DBS), and identifying under which conditions excessive beta oscillations occur. We found that 6-OHDA hemi-PD rats presented an impaired performance in all experiments compared to the sham group, and DBS could improve their overall performance. Across all the experiments and behaviors, the power in the high beta band was observed to be an important biomarker for PD as it showed differences between healthy and lesioned hemispheres and between 6-OHDA-lesioned and sham rats. This all shows that the 6-OHDA hemi-PD model accurately represents many of the motor and electrophysiological symptoms of PD and makes it a useful tool for the pre-clinical testing of new treatments when low β (13–21 Hz) and high β (21–30 Hz) frequency bands are considered separately.

## Introduction

Parkinson’s disease (PD) is a neurodegenerative disorder which affects an estimated 10 million patients worldwide (Dorsey et al., 2018). The disease is characterized by both motor and non-motor symptoms, including decreased and inhibited movements, resting tremor, rigidity, sleep issues, cognitive dysfunction, and depression (DeMaagd and Philip, 2015). It has been shown that neural loss in the nigral dopaminergic inputs to the striatum is one of the main causes of the condition. This leads to a major alteration in the neural activity in the cortico-basal ganglia loop, which adversely affects the ability to make voluntary movements (Brown et al., 2001; Levy et al., 2002; Kühn et al., 2005). In the presence of normal dopaminergic drive, the activity of cortico-basal ganglia loop neurons is largely desynchronized. However, upon the loss of dopaminergic neurons, in idiopathic PD and experimental models of the disease, neurons of the subthalamic nucleus (STN), (internal and external) globus pallidus (GP) and substantia nigra pars reticulata (SNr), lose their independence and show increases in burst firing, and synchronization of activity (Filion et al., 1991; Nini et al., 1995).

The synchronized neural oscillations in the aforementioned areas mostly have frequencies within the beta band (13–30 Hz), a frequency band which has been shown to correlate with bradykinesia and rigidity in PD patients (Kühn et al., 2008). Research to-date suggests that common treatments for bradykinesia, such as levodopa medication or high-frequency deep brain stimulation (DBS), work by suppressing the synchronized beta band oscillations in the cortico-thalamo-basal ganglia circuit (Levy et al., 2002; Kühn et al., 2005; Dorval et al., 2010; Delaville et al., 2015). This suppression is predominantly observed in the STN (Plenz and Kital, 1999; Gatev et al., 2006; Stein and Bar-Gad, 2013) and the motor cortex (Yamawaki et al., 2008) of PD patients. It is, however, important to note that this suppression of neural activity in the cortico-thalamo-basal ganglia circuit only leads to motor improvements in patients who already present deteriorated capabilities, and will instead impair the performance of PD patients whose motor capabilities are within normal limits (Chen et al., 2011).

Adaptive deep brain stimulation (aDBS) is emerging as a promising enhanced treatment for PD which overcomes several limitations of conventional DBS (Little and Brown, 2020). Since physiological biomarkers derived from cortico-basal ganglia loop beta band oscillations are currently the front-runners as control signals for aDBS (Little et al., 2013, 2014; Castaño-Candamil et al., 2017), the development of future control algorithms for this treatment (Hoang et al., 2017; Neumann et al., 2019) will surely benefit from a framework for the systematic testing of these biomarkers using accessible and established animal models of PD.

In this study we present an investigation on the suitability of the unilateral 6-hydroxydopamine (6-OHDA) hemi-PD rat model (Ungerstedt, 1968) as a framework for PD pre-clinical research. The unilateral 6-OHDA rat model simulates certain symptoms of PD by unilaterally injecting the highly specific neurotoxin 6-hydroxydopamine (6-OHDA) into either the medial forebrain bundle (MFB) or the substantia nigra, causing substantial ipsilateral dopamine loss (Ungerstedt, 1968; Henderson et al., 2003). This creates what is known as a hemi-parkinsonian (hemi-PD) rat, where one hemisphere of the rat brain is significantly damaged, and the contralateral side to the lesion serves as an in-animal control allowing for electrophysiological comparisons within the rat (Lundblad et al., 2005). Throughout this paper, lesioned animals are interchangeably referred to as 6-OHDA, hemi-PD, or PD animals. We use the last two names to highlight the model’s capability to simulate several symptoms of PD, though we would like to make clear that the disease model does not capture all the aspects of PD, as will be demonstrated later.

Our work aims to quantify the motor impairments displayed by the unilateral 6-OHDA model. In addition it characterizes the conditions under which an excess in the spectral power of the beta frequency band is exhibited in the neural oscillations of the primary motor cortex (M1) and the subthalamic nucleus (STN) of animals lesioned according to this. To achieve this, a series of tests assessing the different movement capabilities of the animals were performed, including the cylinder, open field, and rotarod tests. Furthermore, the characterization of the lesion’s influence on animal’s motor capabilities, the effect of standard DBS on the improvement of motor impairments and the alterations of beta oscillations were investigated through the above tests as well. Finally, all throughout our study we assessed the suitability of beta power in the STN as a biomarker to control stimulation in aDBS.

## Materials and Methods

All animal procedures were conducted in conformity with relevant institutional rules in compliance with the guidelines of the German Council on Animal Protection. Protocols were approved by the Animal Care Committee of the University of Freiburg under the supervision of the Regierungspräsidium Freiburg (approval G15/031) in accordance with the guidelines of the European Union Directive 2010/63/UE.

### Surgical Procedure

Two groups of adult female Sprague-Dawley rats (290–310 g), consisting of 13 6-OHDA lesioned hemi-parkinsonian rats (PD-group) and 12 non-lesioned rats (sham-group), were used for this study. All animals were acquired from Charles River Laboratories (Germany) and were housed under temperature-controlled conditions in a 12-h light-dark cycle, with access to water and food *ad libitum*. Rats were allowed to acclimate to these conditions for a minimum of 2 weeks prior to any experimental procedures in order to reduce unnecessary stress.

Prior to surgery, all rats underwent at least 7 days of handling to familiarize them with the experimenter. During each surgery, the rats were anesthetized with oxygen (0.15 l/min) and isoflurane (Abbvie, United States). Anesthesia was induced with 4% isoflurane and gradually lowered to 1.5% after placing the animal into the stereotaxic frame (David Kopf, United States). Breathing, reflexes, and depth of anesthesia were monitored throughout the duration of the surgery.

All PD-group animals underwent two consecutive stereotaxic surgeries, with a 2-week recovery time between each surgery. In the first, the 6-OHDA was injected, and in the second, custom-made microelectrodes for stimulation and recording were implanted. During each surgery, holes were drilled at the injection or implantation site, and the dura was resected using a fine needle. The injection canula or electrode was subsequently lowered manually at a rate of approximately 200 μm/s. Four minutes were allowed to pass after each injection, with the needle still inserted, to allow the 6-OHDA to adequately diffuse into the target. After electrode implantation, the skull aperture around the implanted electrode was filled with bone wax. Once in place, electrodes were fixed to a nearby stainless-steel screw anchor (0–80 × 1/8; Plastics One) using a 2-compound dental cement (Palapress; Heraeus Holding GmbH; Germany).

Sham animals (*n* = 12) were subjected to a unilateral MFB injection of saline, following the same procedure as that of the lesion for the 6-OHDA-group (see section “6-OHDA Lesion and Apomorphine Test”). The sham rats were subsequently subjected to an electrode implantation surgery, which followed the same procedure as the implantation surgery of the PD group (see section “Electrode Implantation” for more details). This protocol ensures that the impairment observed in the hemi-PD rats is solely due to the 6-OHDA treatment and not due to the surgical procedures that the rats were subjected to.

### 6-OHDA Lesion and Apomorphine Test

**FIGURE 1:**
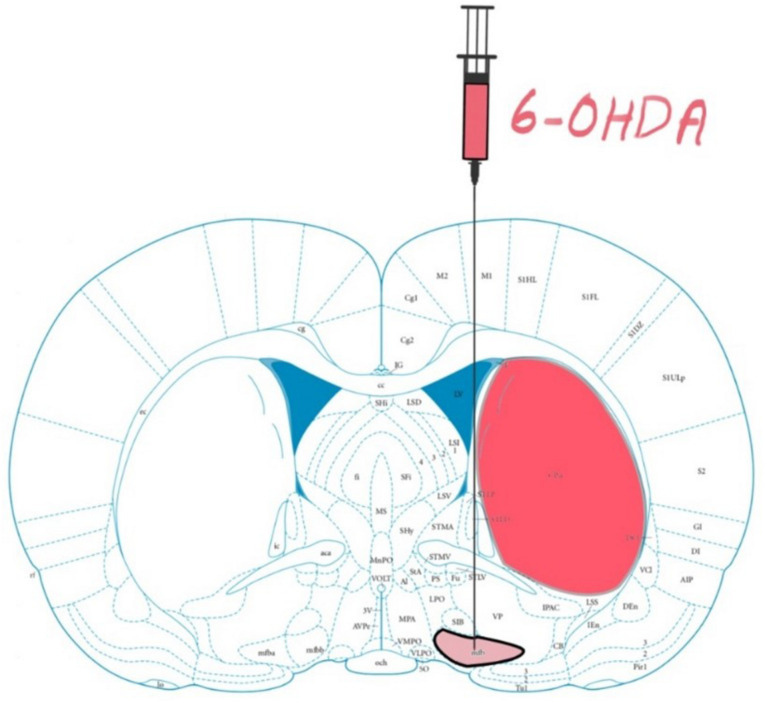
Rat brain atlas; 6-hydroxydopamine (6-OHDA) Parkinson’s Disease (PD) models are made by administering 6-OHDA into nigrostriatal pathway, in this case the medial forebrain bundle (mfb). From 6-14 days post lesioning, the dopaminergic cells in the striatum (Caudate Putamen CPu) degenerate. Figure adapted from Mottaghi (2020).

Animals assigned to the PD group were unilaterally lesioned with a 6-OHDA solution (3.6 mg 6-OHDA, 20 mg ascorbic acid, and 10 ml 0.9% NaCl) injected into the right medial forebrain bundle (Figure 1). Table 1 contains the lesion coordinates, 6-OHDA volume and the injection rate. The Apomorphine rotation test was performed after a recovery period of 2 weeks to test dopamine depletion intensity. Animals were deemed sufficiently lesioned if at least 3 contralateral (to the lesion) rotations on average were observed per minute for 30 min after the subcutaneous injection of a 0.1ml/100 gr. Apomorphine solution (1 mg Apomorphine, 2 mg ascorbic acid acquired from Sigma-Aldrich Chemie GmbH, Germany and 20 ml NaCl).

**TABLE 1:** Lesion coordinates in MFB, quantity of 6-OHDA per coordinate and injection rate.

	AP (mm)	ML (mm)	DV (mm)	Quantity	Injec. Rate
MFB	−4.4 −4.0	−1.2 −0.8	−7.8 −7.2	2.5 μl 3.0 μl	1 μl/min 1 μl/min

### Electrode Implantation

**FIGURE 2:**
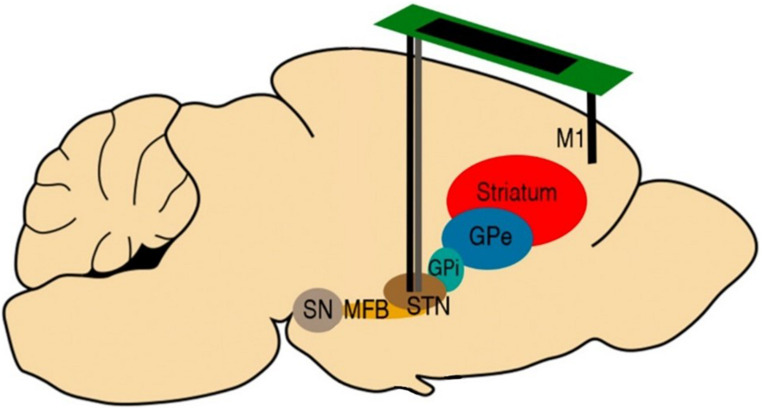
Schematic description of the electrode implantation sites. All rats received bilateral implantations of Platinum-Iridium bipolar recording electrodes (10 mm diameter, black) to both the STN and M1. Rats in the PD group were also implanted with bipolar stimulation electrodes (50 mm diameter, gray) in the STN ipsilateral to the lesion, adjacent to the recording electrodes. Figure adapted from Mottaghi (2020).

All rats received a bilateral implantation of Platinum-Iridium (70% Pt, 30% Ir) bipolar recording electrodes with 10 and 75 μm tip diameter and separation, respectively (Science Products GmbH, Germany) into the primary motor cortex (M1) and STN, to achieve a total of eight recording sites for each rat brain (Figure 2). The coordinates of the implants are available in Table 2. Additionally, rats in the PD group received custom-made bipolar stimulation electrodes, made of intertwined 50 μm Platinum-Iridium (70% Pt, 30% Ir) microwires (Science Products GmbH, Germany). The stimulation electrodes were implanted into the STN ipsilateral to the lesion and adjacent to the recording electrodes (Figure 2).

**TABLE 2:** Electrode implantation coordinates in M1 and STN.

	AP (mm)	ML (mm)	DV (mm)
M1	+2.5	±3.0	–1.6
STN	−3.8	±2.4	8.0

### Animal Experiments

In all experiments, video recordings of animal behavior and electrophysiological recordings of brain signals were collected. Electrophysiological data in the form of local field potentials (LFP, 0.3–300 Hz filtered using a 20th-order Butterworth filter) was recorded from the bilaterally implanted recording electrodes in the STN and M1. Data was collected using a 32-channel wireless head stage (Multi-channel System GmbH, Germany) and an AlphaLab SnR system (AlphaOmega, Israel). Prior to all behavioral tests, an adequate stimulation strength for each animal of the PD group was individually determined by titrating the current amplitude such that explorative behavior was observed, but stimulation-related side effects were not. In this way, we compensated for possible variations in electrode placement and shape. Stimulation was applied at a frequency of 130 Hz using a biphasic rectangular pulse and a pulse width of 65 μs in each of the following behavioral tests. Depending on the behavioral paradigm being tested, DBS stimulation was turned on/off at different points throughout the experiment for a fixed period of time to compare the behavior of the animals during on/off periods.

#### Cylinder Test

The cylinder test measures spontaneous forelimb use, body movement and exploratory activities (Cenci and Lundblad, 2005). Animals were not introduced to the cylinder prior to the experiment in order to test their motivation and capability to explore a novel environment.

**FIGURE 3:**
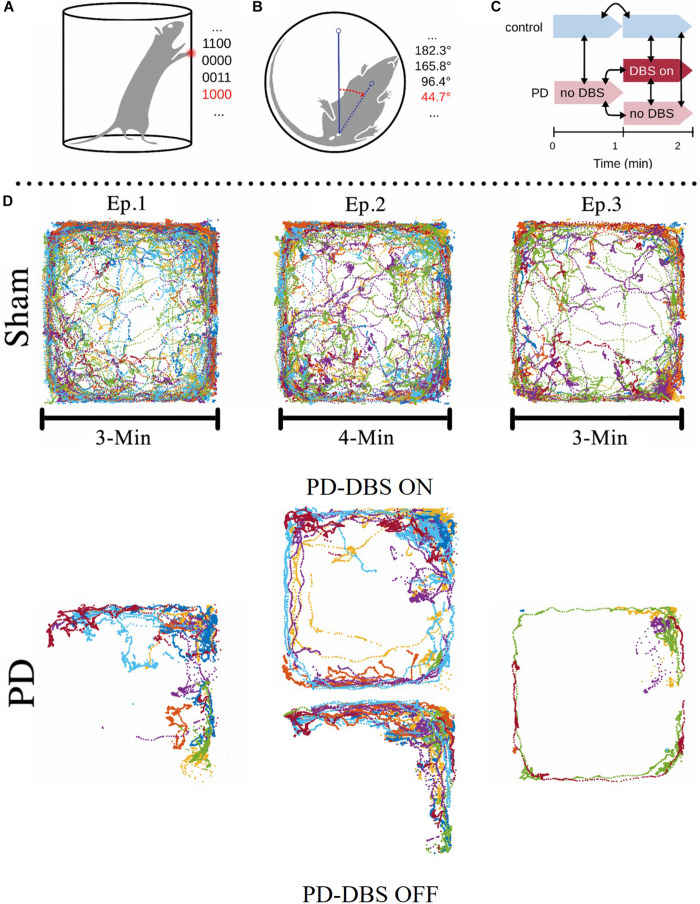
Depictions of the cylinder test and open field test experimental setups. **(A,B)** The body angle of the animal and the position of its forepaws were measured and the latter was recorded using a binary code. The first two digits of the 4-bit code represent whether the forepaws were touching the cylinder walls and the last two represent whether they were touching the cylinder floor (e.g., 1100 = both front paws on wall, 0000 = rearing with no support, 0011 = both front paws on floor, 1000 = left paw on wall and right paw not in contact). **(C)** The animals were monitored for 2 min. A subgroup of PD animals received DBS treatment at the onset of the second minute, while the rest of the PD group remained untreated. **(D)** Open field area, a 74 cm × 74 cm square box, with the exploratory activities of different PD and Sham rats during 10 min superimposed. Note how sham rats explore most of the area available to them (with a tendency to avoid the center which is a natural reaction for rats), whereas unstimulated PD rats remain on the corners and explore much less than sham rats. Notice the significant difference in the trajectories between stimulated PD rats (episode 2, top) and non-stimulated PD rats [episode 1, 2 (bottom) and 3]. This shows that DBS considerably increases the rats’ ability or motivation to explore and corrects major motor impairments. Figure adapted from Mottaghi (2020).

Each rat was placed in a transparent cylinder (acrylic glass, 19 cm diam., 35 cm ht.) for a total of 2 min (see Figures 3A,B). A Logitech C920 HD video camera (Logitech, Switzerland) was used to record the animals from a ventral viewpoint (30fps). Rats belonging to the PD group (*n* = 13) were split into two groups, those receiving DBS during the experiment (PD-DBS ON, *n* = 7) and those which did not (PD-DBS OFF, *n* = 6). Sham rats (*n* = 12) never received DBS. The experimental schedule is shown in Figure 3C.

The observed behavior of all rats was classified into three distinct patterns: rearing, stepping and inactive behavior. During rearing, rats stand on their hind limbs while their forelimbs touch the cylinder wall to maintain balance (Figure 3A). Stepping was defined as episodes where the orientation of the animal’s body shifted > 45 degrees while its forepaws alternated between touching the ground and being held in the air (Figure 3B). Any behavior that could not be defined as either rearing or stepping was classified as inactive.

To quantitatively assess the amount of time spent in each of the three behavioral categories, the distinct positions of the rat’s forelimbs were identified and converted into a four-digit binary code, updated every 0.25s. The first two digits represent the left and right forelimb, respectively, and indicate whether the paws touch the cylinder wall (1) or not (0). Similarly, the last two digits show whether the forelimb paws touch the ground (e.g., 1100 = both front paws on wall, 0011 = both front paws on floor, 0000 = rearing with no support, 0110 = right paw on wall and left paw on floor). The body rotation was also measured (in θ/s) using Tracker video analysis software (©2016, Douglas Brown^1^) combined with a virtual protractor overlay on the video, with the protractor vertex centered on the center of the urinary meatus of the rat. The endpoint of one protractor leg was aligned with the upper chest of the animal while the remaining leg served as a reference for rotation. After collection, the binary forelimb states and rotation measurements were subsequently analyzed in MATLAB (MathWorks, United States).

#### Open Field Test

To measure the animal’s locomotor performance and explorative behavior in a novel environment, an open field test (Seibenhener and Wooten, 2015) was performed. The rats were placed in a featureless square chamber (74 × 74 × 30 cm) and left free to explore for 10 min while their location was tracked using BioObserve Viewer II software (BioObserve GmbH, Germany, 25 fps). The experimental layout, together with the superimposed trajectories of the tested animals, are depicted in Figure 3D. The experiment was divided into three episodes. During the first 3 min (episode 1), no rat (*n* = 25) received DBS stimulation. Next, PD-group animals were divided into two subgroups; one received DBS for 4 min (PD-DBS ON, *n* = 7) while the other did not (PD-DBS OFF, *n* = 6). In the last episode, DBS was turned off for all the PD rats (*n* = 13) and stayed off for the remaining 3 min. Sham rats (*n* = 12) were left free to explore with no DBS for the entirety of the experiment. The environment was cleaned after each trial to prevent any lingering olfactory signals from interfering with behavior. As in the cylinder test, electrophysiological and video tracking data was collected and subsequently analyzed offline.

#### Rotarod

**FIGURE 4:**
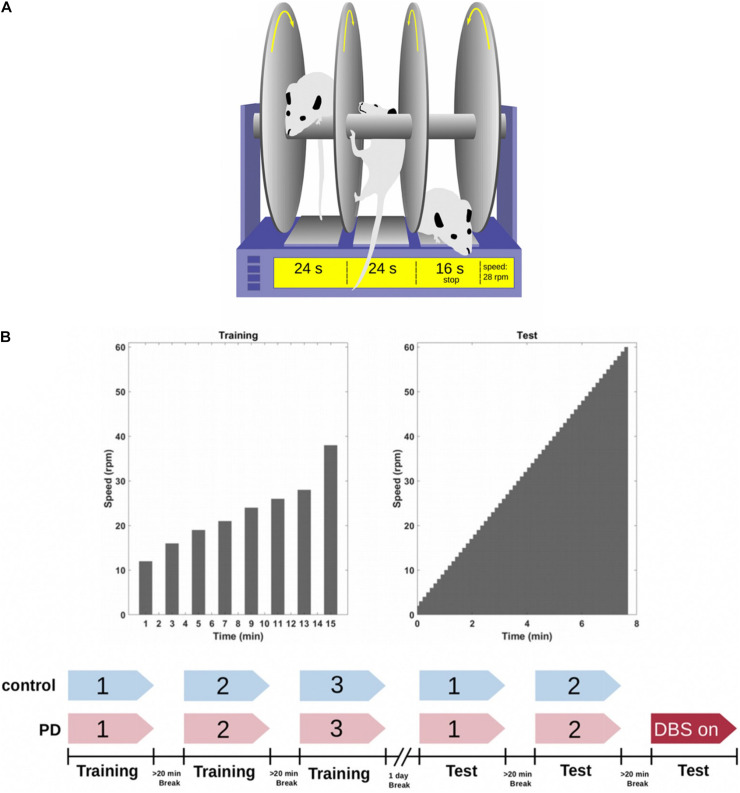
**(A)** The rotarod is a device consisting of a rotating rod on which rats have to walk without falling for as long as they can. A timer records how long the rats walk on the rod before they fall and trip a switch which stops the timer. **(B)** All the animals (*n* = 25) were trained on the rotarod 1 day prior to the test day. They trained for three sessions (with a maximum duration of 60 s) with a 20 min break between each session. On the next day, both the sham and PD group performed two sessions of testing without DBS. A subgroup of the PD rats (*n* = 7) performed a third round while receiving DBS treatment. The other subgroup performed the third round without DBS (*n* = 6). Figure adapted from Mottaghi (2020).

The rotarod test, first described by Dunham and Miya (1957), is widely used to assess the effect of brain injuries or experimental drugs on motor function in rodents (Cartmell et al., 1991; Bohlen et al., 2009). No experienced observer or nominal scoring procedure is required, as the test yields a discretely measurable variable (time or speed) which can be used to quantify motor behavior in an objective manner. To perform this test, the rat is placed on a rotating rod that mimics a treadmill. The rod is suspended high enough over the ground so that the rat is naturally motivated to avoid falling, but low enough to avoid any injuries should a fall occur. The rotational speed of the rod is then gradually increased until, when the rat eventually loses its grip or balance, it falls onto a switch plate beneath the rod. Both the speed of the rod and the time that the animals remained on the rotarod are measured and recorded (Figure 4A).

The experimental schedule spans over 2 days: on the first day, all rats (*n* = 25) were trained on a rotarod (Rat Rota-Rod 47700; UGO Basile S.R.L. Gemonio, Italy) in three sessions with 20 min between each session. The sessions consisted of 8 trials with different velocity settings of the rod (12, 16, 19, 21, 24, 26, 28, and 38 rpm) and a maximal duration of 60s. Each trial was followed by a resting period of 1 min (Figure 4B). On the second day, the rotarod was set to accelerate from 2 to 60 rpm with 1 rpm steps over the course of 7 min. 52 s. All rats performed the test in two sessions with a break of at least 20 min in between the sessions. The PD rats were then split into two groups where PD-DBS ON rats (*n* = 7) performed an additional third session while constantly receiving DBS and PD-DBS OFF (*n* = 6) did the same but without DBS (Figure 4B).

### Electrophysiological Analysis

All of our data analysis was performed in MATLAB 2017a (Mathworks, United States) and Python (Python Software Foundation, CWI). LFP signals were resampled at 1.1 kHz. Power spectral density (PSD) was calculated using Thomson’s multitaper PSD estimate (5 Slepian tapers). In this paper, the right hemisphere of PD-group animals corresponds to the lesioned side, while the left hemisphere is the intact (unlesioned) side. Sham rats only had recording electrodes and hence both of their hemispheres were intact.

The Euclidean Distances (ED) between the averaged PSD’s of different brain regions were used to quantitatively compare electrophysiological behavior across the brain. In our experiments these were used to quantify the difference in beta band power (13–30 Hz) between sham rats and PD rats as follows:

(1) $$ED_{PSD_{sham}}-PSD_{PD}=\sqrt{\sum_{f=13Hz}^{30Hz}PSD{\left(f\right)}_{sham^{2}}-\sum_{f=13Hz}^{30Hz}PSD{\left(f\right)}_{PD^{2}}}$$

The behavioral results and normalized band power were statistically analyzed using a non-parametric Wilcoxon rank-sum test, unless mentioned otherwise. Kendall’s rank correlation coefficient was used to assess the linear correlation between band power and speed.

### Euthanasia and Histology

After having finalized all experimental testing, chronically implanted rats were euthanized with an overdose of isoflurane and were perfused transcardially with a 4% formaldehyde solution (PFA in phosphate buffer). Their brains were removed, post-fixed in PFA for 7 days and stored in 30% sucrose, after which they were cut into coronal sections (40 μm) along the probe’s implantation trajectory with a cryostat (CryoStar NX70, Thermo Fisher Scientific, United States). Sections were collected on glass slides and stored at 4°C until further processing. Tyrosine Hydroxylase TH staining (Primary AB: T-1299 Sigma 0.2 ml −20°C) was then used as a marker to identify the presence (or lack of) dopaminergic neurons and thus assess the success of the lesion. The sections were also used to verify the correct positioning of the recording and stimulating electrodes.

## Results

**FIGURE 5:**
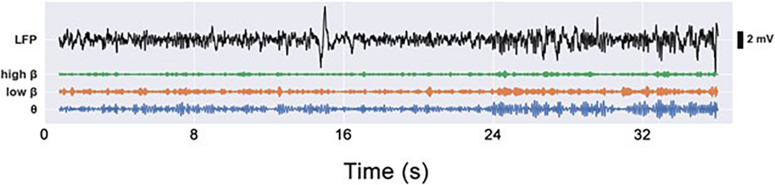
Electrophysiological LFP signals (0.3–300 Hz), as well as video footage, were captured during each experiment simultaneously and were analyzed offline. LFP signals were filtered into G (6–12 Hz), low p (13–21 Hz), and high p (21–30 Hz) frequency bands.

Using a series of different experimental paradigms, we provided an in-depth characterization of the motor impairments caused by the unilateral 6-OHDA lesion of the hemi-PD rat model and investigated the conditions under which excessive beta power can be observed in the STN and M1 of animals lesioned according to this model. For each experimental paradigm we analyzed the behavior and compared the impairments of the PD group with the motor abilities of the sham group. Furthermore, we investigated the differences in neural activity arising from the different behaviors. To this end, the recorded LFP signal (0.3–300 Hz) was filtered into θ (6–12 Hz), low (13–21 Hz), and high (21–30 Hz) β bands (see Figure 5).

### DBS Restores Exploratory Movement Patterns in the 6-OHDA Hemi-PD Rat

The cylinder and open field (OF) tests investigated exploratory movement patterns in both the PD and sham animal groups, since the animals were free to move and explore in both environments. The cylinder environment favored rearing and other stationary activities while the OF challenged their locomotion capabilities.

**FIGURE 6:**
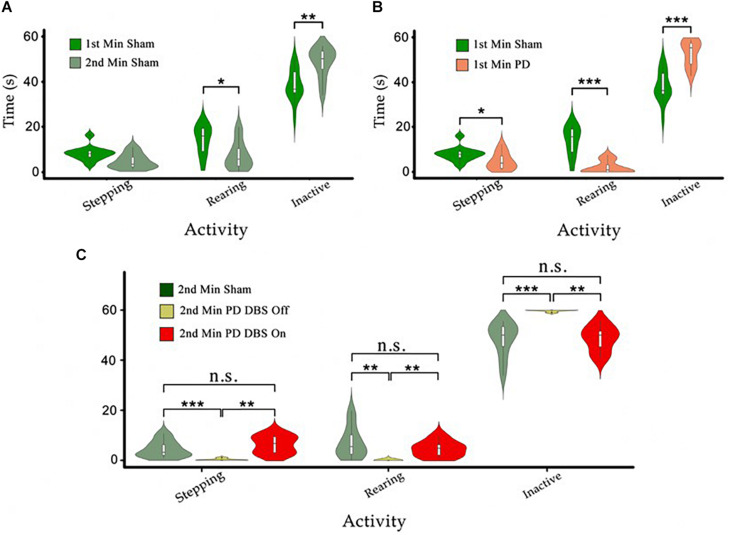
**(A)** The time sham animals spend in stepping, rearing, and inactivity during the first minute of the tests was compared to the second minute. We observe decreases in stepping and rearing behavior and an increase in the time spent in the inactive state. **(B)** Comparison in the same behaviors as in **(A)** between PD and sham rats during the first minute. **(C)** The behavioral patterns of PD-DBS ON, PD-DBS OFF, and sham animals during the second minute of the cylinder test. We see a similar pattern for PD-DBS ON and sham rats and a drastically impaired pattern in PD-DBS OFF rats, which spend nearly all of their time in the inactive state. Level of significance (*p < 0.05, **p < 0.01, ***p < 0.001, and n.s. stands for not significant). Figure adapted from Mottaghi (2020).

In the cylinder test, the time that each animal spent in each of the 3 behavioral categories (i.e., rearing, stepping, and inactive) was measured and analyzed individually for each category. To establish a baseline behavioral pattern, the performance of the sham rats during the first minute of the experiment was compared to their performance during the second minute; thus focusing on any behavioral changes resulting uniquely from the time spent in the cylinder. A significant decrease in the sham rats’ rearing time and a significant increase in the duration and frequency of their inactive episodes was observed from this comparison (p_rear_ < 0.05, p_inactive_ < 0.01). However, the difference in the time spent stepping was insignificant (see Figure 6A). This shows that there is a natural tendency for the rats to explore less the longer they spend in the cylinder environment. These results are useful to gauge the scale of the natural decrease in exploratory behavior over time, allowing a more accurate estimation of the effects caused by the 6-OHDA lesion.

A similar comparison between the PD and sham groups over the first minute shows that PD rats spent significantly less time stepping and rearing, and instead spent more time in the inactive state (p_stepping_ < 0.05, p_rear_ < 0.001, p_inactive_ < 0.001) (Figure 6B). The first minute differences in the time spent stepping between PD and sham rats are slightly more pronounced than comparable first to second minute sham rats’ efforts (“Stepping,” Figure 6B vs. Figure 6A). The differences in time spent rearing and in the inactive state are however much more pronounced between PD and sham rats than among sham rats only (“Rearing” and “Inactive,” Figure 6B vs. Figure 6A).

To evaluate the effect of DBS on the behavior of PD rats, the PD-group was divided into two subgroups: one PD subgroup (*n* = 7, PD-DBS ON) was exposed to STN-DBS at the onset of the second minute of the experiment, while the other subgroup remained untreated (*n* = 6, PD-DBS OFF). The results indicate that PD-DBS ON rats exhibited a similar behavioral pattern to the sham group during the second minute, whereas PD-DBS OFF rats exhibited significantly less stepping (p_Sham– PD–DBSOFF_ < 0.001 and p_PD–DBSON –PD–DBSOFF_ < 0.01, respectively) and rearing (p_Sham–PD–DBSOFF_ < 0.01 and p_PD–DBSON –PD–DBSOFF_ < 0.01, respectively), and instead spent significantly longer periods of time in the inactive state (p_Sham–PD–DBSOFF_ < 0.001 and p_PD–DBSON – PD–DBSOFF_ < 0.01, respectively) compared to the sham and PD-DBS ON groups (see Figure 6C).

The data from the OF test showed similar results to that of the cylinder test. This data was analyzed for all groups by dividing the tracking data into three episodes: (1) the first 3 min of the experiment without electrical stimulation; (2) minutes 4 to 8, where only the PD-DBS ON subgroup (*n* = 7) received DBS, and the rest of the groups continued untreated; (3) the last 3 min, where once again no DBS was applied to any of the groups. Subsequently, we compared the behavioral patterns exhibited in each of the episodes across groups.

Figure 3D illustrates tracking data for sham and PD group animals in each episode. Similar to the cylinder test, it can be seen that the sham rats’ exploration drive declined as time progressed. Figure 3D also shows that sham rats explored significantly more than PD-group rats, where PD animals tend to move less and like to stick to the walls of the arena. Although DBS increased the extent of exploratory behavior of PD rats in episode 2, when it was removed during episode 3, PD animals returned to their mostly inactive behavior.

**FIGURE 7:**
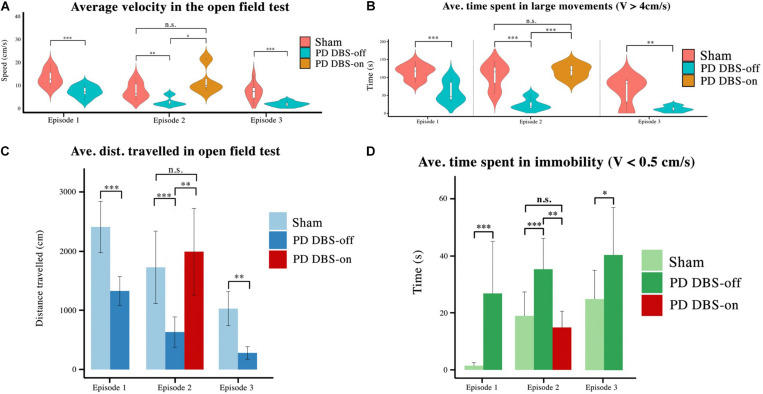
Four behavioral features of the OF test were analyzed using the animals’ tracking data. **(A)** The average velocity, **(B)** time spent in large movements, **(C)** distance traveled, **(D)** and the time spent in immobility were selected as variables to compare across groups. It is shown here that unstimulated animals exhibit lower velocity, distance traveled, and time spent in large movements than stimulated or healthy animals, and instead spend more time in immobility. However, the subgroup that received DBS exhibits significant exploratory improvements and a significant reduction in their immobility spells. Level of significance: (*p < 0.05, **p < 0.01, ***p < 0.001, and n.s. stands for not significant). Figure adapted from Mottaghi (2020).

In order to quantify and statistically compare these behavioral differences, four parameters were considered: average velocity (see Figure 7A), average time showing large movements (LM) (speed > 4 cm/s, t > 2 s) (see Figure 7B), total distance traveled (see Figure 7C) and average time spent immobile (speed < 0.5 cm/s, t > 2 s) (see Figure 7D). Sham rats showed a decrease in average velocity, average time spent in LM and distance traveled if we compare the results from the first and third episodes. On the other hand, the average time sham rats spent immobile increased throughout this timespan. This corroborates habituation observed in the cylinder test.

During the first and third episodes (where DBS was not applied to any animal), significant differences in all four parameters were seen between the sham and PD groups (p_speed_, p_LM_, p_tr.dist._, and p_immob._ < 0.001). In the second episode, no significant difference was observed between any of the measured parameters for sham rats and the PD-DBS-ON subgroup, however the PD-DBS-OFF subgroup exhibited significantly less exploratory behavior in comparison.

The behavioral results from both the cylinder and OF tests hence show that we can reestablish severely impaired movement capabilities to close-to-healthy levels through the use of STN-DBS on subjects of the hemi-PD rat model.

### Distinct Movement-Dependent PSD Profiles in 6-OHDA Hemi-PD Rats

Having characterized the behavioral differences between the sham, PD, and DBS groups, we next examined how these differences were reflected on a neural level. To relate the animals’ behavior with the measured neural activity, the digitized behavioral patterns of the animals’ forelimbs in the cylinder test were synchronized with the time-frequency power analysis of the electrophysiological recordings in order to identify possible movement-dependent patterns. A digitized value > 0100 (4 in its decimal representation) encodes for a rearing position as here at least one of the animal’s forelimb paws is touching the wall (1100 = both front paws on wall, 0000 = rearing with no support, 0011 = both front paws on floor, and 1000 = left paw on wall and right paw not in contact).

**FIGURE 8:**
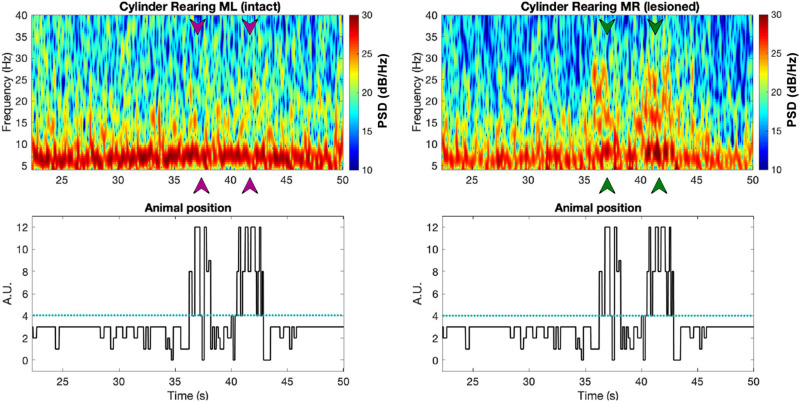
The top two plots show spectrogram examples from the intact (left) vs. lesioned (right) hemispheres captured from bilateral M1 of a PD rat (R21), with the digitized behavioral pattern shown below each spectrogram. Note that the arrows in the spectrograms point at the times in which the animal was rearing, as can be seen from the forelimb position plot below (green and purple correspond to the lesioned and intact hemispheres, respectively). Figure adapted from Mottaghi (2020).

**FIGURE 9:**
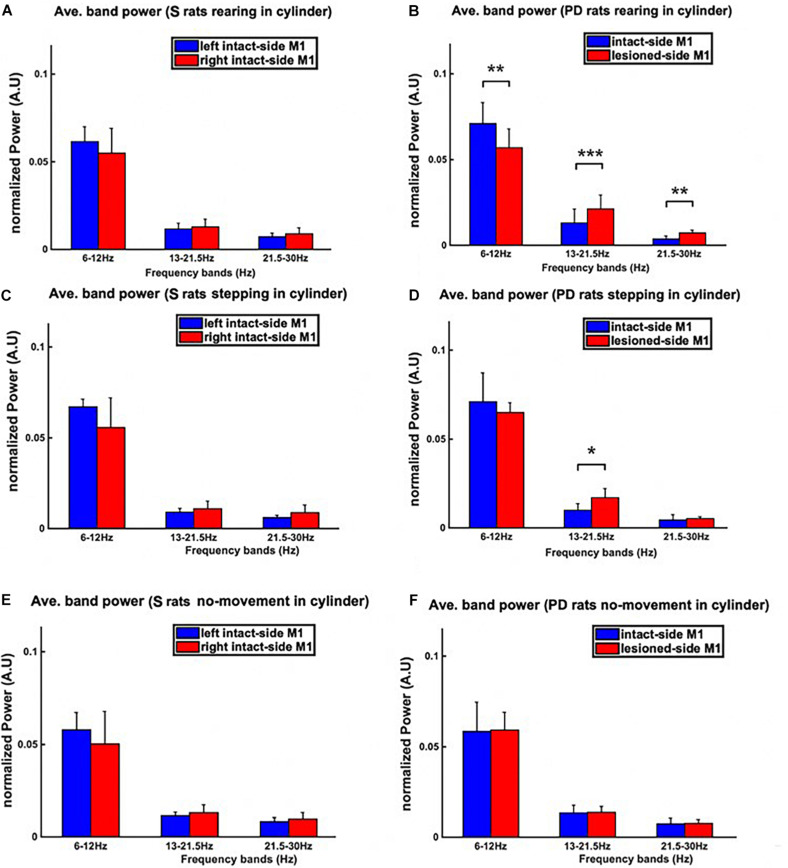
The power spectral density (PSD) during the three behavioral patterns of the cylinder test (i.e., rearing, stepping, and no-movement) was analyzed. The PSD was averaged over theta, low beta, and high beta bands measured from both hemispheres, resulting in three bins, one for each band. The averaged-PSD comparison of the right vs. left M1 of sham rats is shown in the left column **(A,C,E)**, whilst the same comparison for PD rats is shown in the right column **(B,D,F)**. **(B)** During rearing in PD rats, both the low and high beta power from the lesioned hemisphere (right hemisphere) is significantly higher than in the intact hemisphere. **(D)** During stepping episodes, however, only the power in the low beta band is significantly higher in the lesioned than in the intact hemisphere. **(F)** No significant differences between hemispheres can be observed for any band during inactive episodes in PD rats. **(A,C,E)** Similarly, no significant differences are found by comparing the power in PD animals to that of sham group animals for inactive episodes. Level of significance: (*p < 0.05, **p < 0.01, and ***p < 0.001). Figure adapted from Mottaghi (2020).

Figure 8 shows spectrograms from an electrophysiological recording from both the M1 of the unlesioned and lesioned sides of one exemplary PD rat, synchronized with a plot showing the rat’s progression through different forelimb positions. The lesioned hemisphere in this PD rat shows a strong increase in beta power (13–30 Hz) compared to its unlesioned (intact) hemisphere in moments when the rat was entering the rearing position (see arrowheads below spectrograms, Figure 8). A similar analysis applied across all rats (see Figure 9) confirms the findings of Degos et al. (2009) who also observed an excess in beta band power in the lesioned hemisphere of rats from the unilateral 6-OHDA model. The correspondence of the peak in beta power with rearing episodes shows that the model is affecting neural activity during movement planning and initiation, which could explain the tendency of PD rats to remain in the inactive state (see Figure 6B and Figure 7D).

For both sham and PD rats, we also calculated the average band power within the frequency bands of interest [i.e., theta (6–12 Hz), low beta (13–21 Hz), and high beta (21–30 Hz)] for signals corresponding to the three behavioral patterns of the cylinder test (see Figure 9). The results show distinct low and high beta band power differences between intact and lesioned hemispheres during rearing, significant differences only in low beta band power during stepping and no significant differences at any of the three frequency bands during inactive episodes. As a control, we also looked at the differences in the band power between the two intact hemispheres of sham rats, finding no significant differences. This hence confirms that the differences in band power for PD rats were due to the lesion.

These results show us that the differences in the spectra of electrophysiological recordings from the left and right M1 of PD and sham rats change depending on the behavior of the rats. In particular during active episodes (Figures 9A–D), the spectra of intact and lesioned hemispheres differ more than in no-movement episodes (Figure 9F). Motor symptoms of PD are apparently related to an alteration of LFP spectra. In addition, the differences in spectra between lesioned and intact hemispheres were preferentially visible in the beta band (both low and high), hinting at the beta band’s importance as a potential indicator for the presence of PD symptoms.

### Excessive Beta Power Is More Prominent in M1 Than in the STN

The prominence of excessive beta power in the lesioned hemisphere of PD rats was compared between recordings from M1 and the STN of PD and sham rats performing the cylinder test. To this end, we computed the PSD of M1 and STN recordings for all animals during rearing and stepping episodes – where the difference in beta power between intact and lesioned hemispheres had been found to be significant.

**FIGURE 10:**
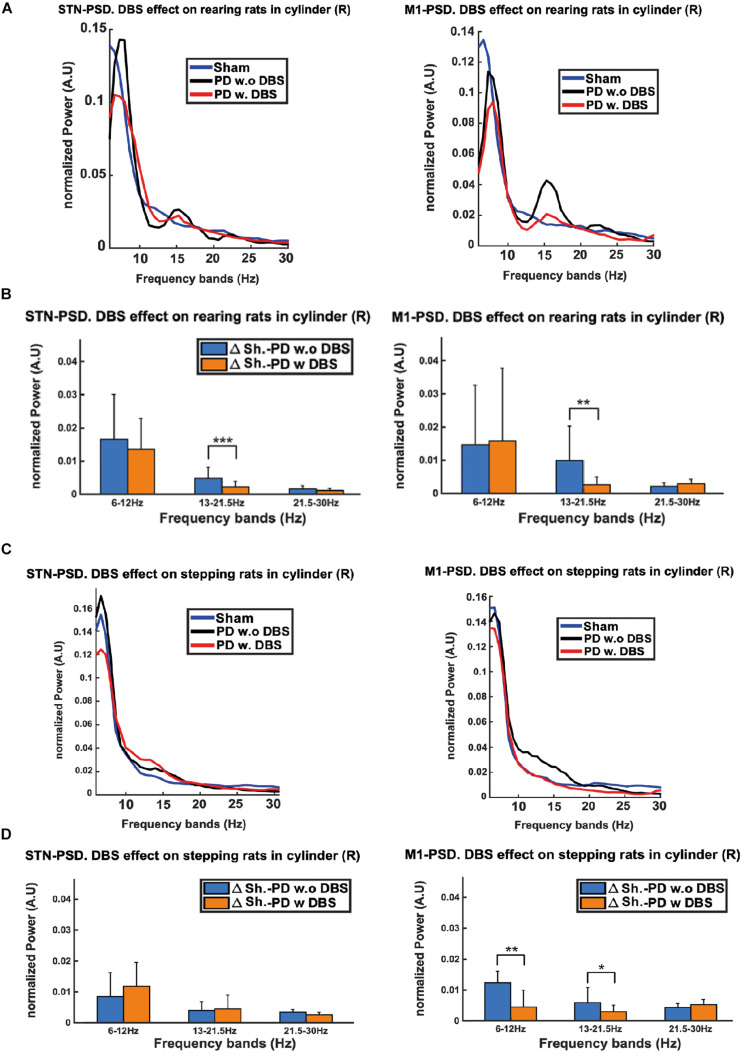
The excessive beta power of PD rats during stepping and rearing was compared between STN and M1 regions. **(A)** Although excessive beta can be seen in both regions, the M1 exhibits a more pronounced excess during rearing as compared to the STN. **(C)** The beta power excess during stepping only becomes apparent for M1 and not for the STN. We also observe that the administration of DBS lowers the beta excess power in both rearing and stepping episodes. **(B,D)** Euclidean Distance (ED) was used to quantify the differences between the PSDs of PD DBS-ON vs. sham and PD DBS-OFF vs. sham rats. The low beta band shows significant decreases in the ED’s upon stimulation, which can be observed in both the STN and M1 during rearing, but only in M1 during stepping. Level of significance: (*p < 0.05, **p < 0.01, and ***p < 0.001). Figure adapted from Mottaghi (2020).

Figure 10 illustrates the PSD analysis of recordings from the STN and M1 of the lesioned hemisphere of PD rats during DBS-OFF and ON episodes, as well as from the corresponding intact regions of the sham group. In order to quantify the difference between the PSD’s from different regions, the Euclidean Distances (ED) between the averaged PSD’s were calculated as in Eq. 1.

The PSD plots show how, in both rearing and stepping episodes, the excess in beta power of the DBS-OFF recordings from the lesioned regions is less prominent in STN (Figures 10A,C left) than in M1 (Figures 10A,C right). In addition, we observe that the application of DBS suppresses this excessive beta power in both regions during these episodes. The results of the ED analysis show in rearing episodes (compared to no stimulation) DBS substantially lowers the ED’s in the low beta band for both the STN and M1 (Figure 10B). A similar analysis for stepping episodes shows a significant reduction in the ED’s for the low beta band only in M1 (not in the STN, Figure 10D).

As the LFPs from M1 had more clear and marked differences between the electrophysiological signals coming from lesioned and healthy hemispheres, this indicates they may be better suited for biomarker applications. Furthermore, differences in the M1 signals were also observed in a wider range of behaviors (i.e., both rearing and stepping) compared to the STN signals. In addition, in line with the results from the previous section, differences in the beta band power between the lesioned and healthy hemispheres were more pronounced as the animals’ activities become more complex and intensive. Finally, the therapeutic effects of DBS also returned the beta band power of PD rats close to the levels of healthy rats.

### The Low Beta Band Is Related to Locomotion

**FIGURE 11:**
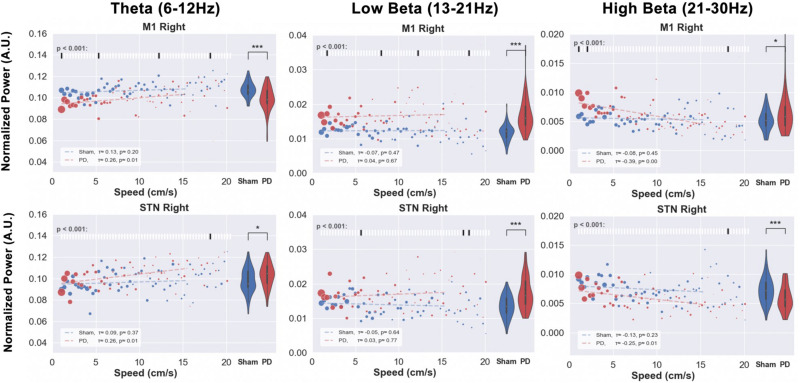
Speed-power relationship was analyzed in order to investigate the correlation between behavioral impairments and electrophysiological indicators. The locomotion speed of each animal was computed using the tracking data. Electrophysiological signals from M1 and STN of the same timestamps were filtered in theta, low, and high beta bands. The scatter plots above illustrate the median value of the band power at each specific speed (red for PD and blue for sham). The size of each point represents the relative amount of data (number of animals exhibiting that speed) per point. The violin plots on the right side of each figure represent the distribution of power over all the speed bins. Kendall’s rank correlation coefficient (x) was computed to assess the correlation between speed and power band values, as shown in the white box in the bottom-left corner of each subfigure. Significance tests between PD and sham band power values at each speed were performed and are shown in the raster plot along the top of each subfigure. Note: Except for the low beta band, PD animals’ LFP power does not exceed healthy animals’ power with the exemption of high beta power at low speeds in the M1. Level of significance: (*p < 0.05 and ***p < 0.001). Figure adapted from Mottaghi (2020).

Next, in the context of bradykinesia in PD, we investigated the correlation between movement speed and spectral power in sham and PD rats. This was done selecting time periods involving locomotion from the OF test. For each locomotion speed in the range 1–16 cm/s (1cm/s resolution), we analyzed LFP signals recorded from M1. Due to low sample points, higher speeds were excluded from the analysis. In order to assess the effect of movement speed on the power in the different frequency bands, the average band power calculated over 5-s windows was determined at each speed. Violin plots on the right side of each subplot in Figure 11 represent the distribution of the power in each band. Additionally, the raster plots placed along the top of Figure 11’s subplots indicate statistical significance for the difference in power between the sham and PD group (blue and red, respectively) at each speed.

Comparing the normalized power within theta, low, and high beta bands at each observed speed between healthy (blue) and PD (red) animals revealed little differences at individual speeds, but significant differences overall. Compared to sham group rats, PD rats demonstrate lower theta and higher low beta power.

The results of this experiment tell us that the speed of free movement is hardly reflected by the band power in both sham or hemi-parkinsonian rats. However, overall utilization of spectral power differs between healthy and lesioned animals (violin plots), but only in the low beta band the PD animals’ power appears more pronounced than in the healthy animals (correlation lines). In other bands the verdict is not that clear and hardly any of the differences in speed-power-pairs shows a significant difference. As a useful indicator should exhibit some kind of specific feature, this casts doubt on the beta band power to be suitable for revealing acute impediments in the movement of PD animals upon which to trigger a stimulator.

### DBS Restores Locomotion and Balance During Forced Movements

**FIGURE 12:**
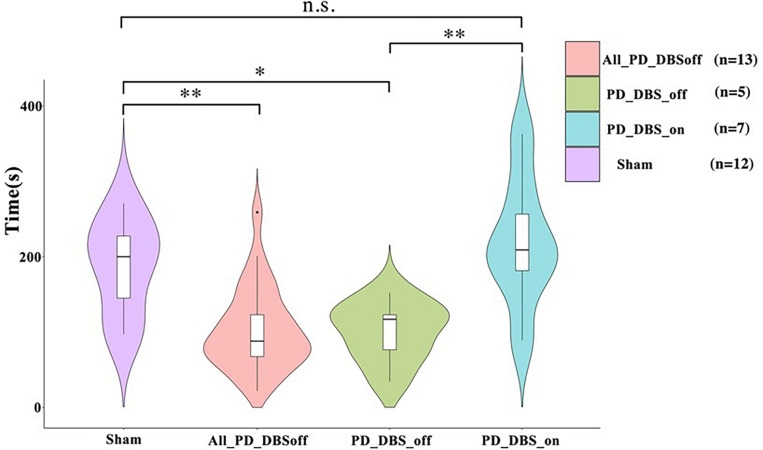
The rotarod test challenges the motor and balance capabilities of animals by forcing them to move at the speed of the elevated rotating rod they are placed on. All the animals underwent two test sessions while no DBS treatment was applied. A subgroup of PD animals performed a third test session while receiving DBS (*n* = 7), whereas the rest of the PD group performed the third round while DBS was off (*n* = 6). The maximum time and speed each animal could remain on the rotarod was recorded and compared across groups. The performance of the PD and sham group during the first two rounds were compared (sham vs. All PDDBSoff). The PD group remained a significantly shorter period of time on the rotarod compared to the sham group. As for the third round, the performance difference between the PDDBSon and sham animals was not significant. However, the performance of the PDDBSoff subgroup was significantly worse compared to the sham and PDDBSon. Level of significance: (*p < 0.05, **p < 0.01, and n.s. stands for not significant). Figure adapted from Mottaghi (2020).

Both the cylinder and OF tests assessed exploratory behavior, allowing the animal to start and stop movements freely. It was shown in Figures 6, 7 that PD animals tend to explore less than sham group animals. This motivated us to challenge their locomotion capability further by employing the rotarod test, in which the rats were forced to initiate movement and continue to walk to avoid falling. We measured the maximum speed and time that each rat was able to stay on the rotarod and compared results across groups (see Figure 12).

As no significant difference was found between the first and the second round of trials in the sham group, we compared the results of any of the two sham rounds to the other trials. Overall, we found that PD rats stayed a significantly shorter (on average 100 s vs. 200 s for sham) on the rotarod compared to the sham group when no DBS was applied. However, remarkably, the administration of DBS greatly increases the time spent on the rotarod for PD rats (*n* = 7) as compared to PD rats without DBS (*n* = 6). Moreover, no significant differences were evident between the sham and PD-DBS group.

These results demonstrate that the movement capabilities of lesioned animals were also impaired for forced movements involving balance, and that DBS is capable of restoring performance back to healthy levels.

### Impairment of Movement Initiation in Hemi-Parkinsonian Rats Is Accompanied by Alterations in Frequency Band Power

**FIGURE 13:**
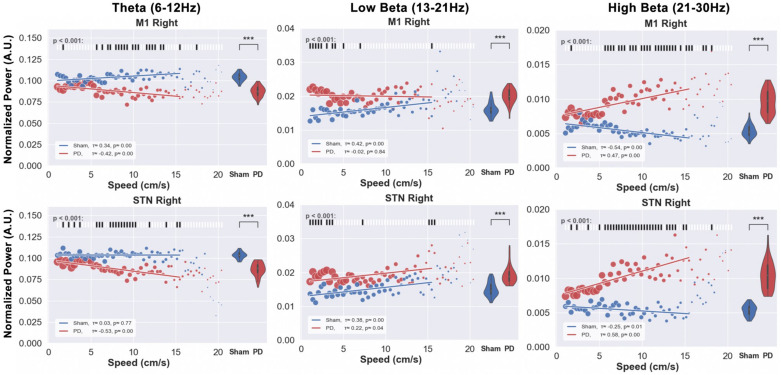
The relation between speed and band power in forced movements on the rotarod was investigated for PD and sham rats. The speeds of the rotarod as well as the electrophysiological signals (M1 and STN) of the same time stamps were plotted for theta, low, and high beta bands. Each data point represents the median of the band power measured at each speed. The size of each point represents the relative amount of data per point. The distribution of the power, over all the speeds is represented by the violin plots on the right side of each subfigure, and the results from the significance test of the difference between the PD and sham band power, at each speed are illustrated in the raster plots above the figures. The theta power, measured from both the right STN and M1 regions over all the speeds (1 16 cm/s) are significantly lower in PD rats compared to the sham group, while both low and high beta power showed higher values for PD compared to sham rats. Unlike the results from the OF test, the results from the STN are in line with the results from M1. Additionally, the low beta band shows stronger significant differences between PD and sham speed-power pairs only at lower speeds (<4 cm/s) while theta and high beta bands exhibit significant differences at higher speeds (>4 cm/s). Level of significance: (***p < 0.001). Figure adapted from Mottaghi (2020).

Finally, we examined how power in different frequency bands related to the performance of the animals in the rotarod test (Figure 13). In the examined theta and beta bands, significant differences were exhibited between the speed-power pairs of PD and sham group animals (see raster plots). The signal power was significantly lower for the PD rats in the theta band and higher in the two beta bands for all speeds as compared to sham rats. Interestingly, significant differences between the power at each speed in the low beta bands were present only during the early phase of the experiment, and disappeared when the speed reached 4 cm/s. The theta and high beta bands instead show strong differences at higher speeds. Therefore, the speed determined whether there was a difference in the speed-power pairs between the groups, which we did not see in the analysis of the OF test (Figure 12). Moreover, contrasting with the results from the OF test, the analysis of recordings from the STN showed patterns similar to those from M1.

## Discussion

Closed-loop or adaptive DBS (aDBS) is hoped to ameliorate PD patient’s symptoms by turning on the stimulation only when needed to reduce PD’s motor symptoms. Clearly, a reliable indicator is needed to trigger the stimulating device and excessive beta-band activity was proposed for that purpose (Little et al., 2013).

Enhanced beta oscillatory activity throughout the cortico-thalamo-basal ganglia loop has been repeatedly reported in PD patients and pre-clinical animal models of the disease (Levy et al., 2002; Brown, 2006; Kühn et al., 2008; Dorval et al., 2010; Delaville et al., 2015). Studies show that it is mainly related to bradykinesia and akinesia symptoms and is thought to be an antikinetic feature of the disease. Dopamine replacement therapies (Heimer et al., 2002; Levy et al., 2002; Williams, 2002; Priori et al., 2004; Sharott et al., 2005) and DBS treatments (Wingeier et al., 2006) have been observed to lower its severity.

In an earlier study of a preclinical PD model, Degos et al. observed such an excessive increase of beta band oscillations in the motor cortex of awake PD rats, unfortunately after the bradykinetic/akinetic symptoms of their model became apparent (Degos et al., 2009).

In the present study we provide a deep behavioral and electrophysiological characterization of the 6-OHDA rat model of PD using three different behavioral paradigms. In each we evaluated motor symptoms, potential electrophysiological indicators, and the efficacy of DBS at recovering motor impairments. Whereas most research with PD models focuses on the basal ganglia’s effect (Dorval and Grill, 2014; Anderson et al., 2015; Hoang et al., 2017) we included LFP recordings from the motor cortex into our data set. Hence our results provide new insights both on the 6-OHDA model as on the quest for a reliable biomarker for aDBS. This is of particular interest as the beta power already has been contested in literature (Swan et al., 2019).

Motor impairments observed in the model (less exploration, slower locomotion speeds, longer immobility) can be linked to bradykinesia and akinesia, while no resting tremor was observed in any of the PD animals in our study which agrees with previous reports on the model (Asakawa et al., 2016). We noticed that excessive beta power was not measured during inactive episodes, corroborating results published by Degos et al. (2009). Similarly (Degos et al., 2009; Asakawa et al., 2016) our hemi-PD animals expressed less exploratory behavior as compared to healthy animals in the cylinder, OF and rotarod tests. We found that DBS treatment improved the performance of hemi-PD animals across all the tested experimental settings, suggesting an analogous mode of operation of DBS in rats to that of humans.

Among the behavior evaluated in unrestrained movement experiments, rearing showed the most prominent increase in the beta band power in lesioned hemispheres. In contrast, we observed when the animal was forced to move on the rotarod, at all speeds, the excessive beta power was detected more easily than during self-guided movements. We speculate that staying atop the rotating rod required better synchronized and more widespread brain networks to be activated thus contributing to a stronger signal in the beta band.

Interestingly, when analyzing the low and the high beta bands separately, we observed in the lesioned hemisphere a cross-over in significance levels at around 4 cm/s forced speed. Below that critical speed, the low beta power was higher in lesioned than in healthy animals, whereas above 4 cm/s high beta is presented stronger by PD animals. This is in line with the literature which assumes high beta band power to be an important indicator for cortico-subthalamic interactions, strongly affected by PD (Lalo et al., 2008; Hirschmann et al., 2013; Cao et al., 2019). With an increase in forced speed high beta energy increases both in the M1 and the STN as compared to the high beta contribution in healthy animals. This divergence is not observed in self-motivated movements.

Low beta power on the contrary is attributed to slower walking behavior and freezing in humans (Singh et al., 2013). A more prominent low beta contribution can be seen in PD animals during the slow phases of the forced motion experiments. Self-motivated movement doesn’t show such a clear distinction. The low-frequency effects of the 6-OHDA model were studied in Alberico et al. (2017), where it was shown that increased delta field potential power correlates with dyskinesias in 6-OHDA-lesioned mice. This low-frequency effect agrees well with our hypothesis that the interference with lower frequencies in both human PD and the 6-OHDA model leads to the disruption of slow movements or movements made when stationary. Although Alberico et al. (2017) were measuring activity in the striatum to demonstrate the correlation, the projections between the STN, M1 and striatum could explain why we have similar findings.

This difference shows that, depending on the speed and the involvement of additional brain circuits, a generalized beta band power tends to ignore important and subtle differences in its subbands. Across all the experiments and behaviors, the power in the high beta band was observed to be an important indicator in PD animals as it showed differences between healthy and lesioned hemispheres and between PD and sham rats.

Previous studies used behavioral tests in 6-OHDA hemi-PD rodent models and quantitatively evaluated the severity of PD symptoms, to investigate the effects of novel therapeutic interventions and to gain insights into PD pathophysiology (Iancu et al., 2005). However, like other neurotoxic models, a limitation is the acute neurodegenerative property of the 6-OHDA model, as it lacks the progressive, age-dependent effects of PD. Nor does it cover the occurrence of Lewy bodies found in humans (Potashkin et al., 2010). We also observed that the 6-OHDA model does not simulate resting tremor, an important symptom of PD in humans. Thus no salient features were detected in signals recorded during periods of inactivity, which would be crucial as aDBS trigger.

The main findings of our experiments were that the 6-OHDA PD model emulates the motor and balance impairments of PD in both free and forced motion and that STN-DBS is well suited to restore animals’ impaired capabilities back to healthy levels. We also discovered that excessive beta power is prominent in PD rats in all experimental settings i.e., in free and forced motion at all speeds – except when animals remain immobile. An analysis between speed and spectral power further revealed significant correlations only in forced movement which were positive in the beta band and negative in the theta band. We also noticed that excessive beta power was more prominent in high beta and M1 than in the STN.

When generously projecting our results from an animal model of PD toward treatments of human patients with adaptive DBS, we advocate to take contextual information of the surroundings into account. Self-paced walking may produce different LFP signals than walking on a treadmill. In addition, control algorithm might strongly benefit from more than one recording location in the brain, as our results showed differences in recordings from STN (the usual implant locationin humans) and the motor cortex.

All in all, we want to express our hope that the use of a better validated animal model might prove beneficial for the treatment of PD patients.

## Data Availability Statement

The raw data supporting the conclusions of this article will be made available by the authors, without undue reservation.

## Ethics Statement

All animal procedures were conducted in conformity with relevant institutional rules in compliance with the guidelines of the German Council on Animal Protection. Protocols were approved by the Animal Care Committee of the University of Freiburg under the supervision of the Regierungspräsidium Freiburg (approval G15/031) in accordance with the guidelines of the European Union Directive 2010/63/UE.

## Author Contributions

SM designed the experiments and performed the surgeries. SM, SK, DB, SL, RM, MW, and CK contributed to the experimental performance. SM and DB analyzed the results. CM performed the histology. RS and UH supervised the experimental and analysis work. All authors contributed to manuscript revision, read and approved the submitted version.

## Conflict of Interest

The authors declare that the research was conducted in the absence of any commercial or financial relationships that could be construed as a potential conflict of interest.

## Publisher’s Note

All claims expressed in this article are solely those of the authors and do not necessarily represent those of their affiliated organizations, or those of the publisher, the editors and the reviewers. Any product that may be evaluated in this article, or claim that may be made by its manufacturer, is not guaranteed or endorsed by the publisher.

## Footnotes

1
https://physlets.org/tracker
